# Fistula beneath the Chin, of Four Year’s Standing

**Published:** 1882-03

**Authors:** 


### FISTULA BENEATH THE CHIN,

### OF FOUR YEAR'S STANDING.

CASE 2.—The lady before you has, you see, a little sore beneath the chin, a trifle to the right of the median line; this sore has persisted, in spite of much treatment, for a period of four years.

A sore, to defy direct treatment, must have something as a support back of it. This sore, I am sure, has something back of it. I have met, in my experience, with many looking like it, but never one where a directly immediate medication applied to its face could, by any possibility, amount to anything in the way of cure. A moment and you will understand this.

The sore before us is the outlet of a fistulous track. It is not at all a thing having meaning in itself; it exists in some other thing, for which we at once make search.

With this silver probe I enter the fistula; it leads, as I presumed we would find, directly to the bone. Here we may pass to the mouth. The patient wears artificial teeth, full sets above and below. Removing the lower set there is nothing of unhealthy character to be seen.

I commit myself, gentlemen, by saying that I have never met with a fistulous sore like the one before us, where a tooth, or the root of tooth, was not at the bottom of it. If here there is found no dental origin, I have to note my first exception.

Confident in my experience, I make a cut along the gum. I dissect the gum from the bone. Convict me, if you please, for a mistake. I confess I never exposed a healthier looking bone. I dismiss the patient that the wound may heal; you shall see the lady later.

* * * This is the patient who, as you will recognize, seemed to convict us of a mistake a few days back. To-day we are to clear up the matter. Ten years back, as I have learned, an operation was performed on this jaw by an eminent surgeon of a distant city, for cure of the condition as we here find it. The bone was exposed and scraped. The result was nil.

The patient being etherized, I make a cut along the shade-line of the neck, directly to the jaw. Let us dissect away, for a little distance from either side of the fistula, the overlying tissue. * * Here is health again, nothing but health. The facial side of this jaw-bone is perfect. I will, with extreme delicacy, expose a little of the neck aspect. Here is a small hole. I stick a steel pin into it to make the location. This hole leads to the meaning of the external sore. What I shall do, is the only thing to be done. I shall follow the lead of the hole and see where it goes. Please note how I shall follow the lead. I pick up the penholder-looking instrument, which you all know as the hand-piece of our surgical engine. My assistant, as you see, has armed it with a burr. Now we will put it in motion. It appears as still as though it were lying untouched upon the table, yet at this moment the burr is making ten thousand revolutions to the minute. It is an instrument of magical delicacy. See what it can do, and how it does what it can do.

*Operation.*—With a few touches, as one would manage a pen, the opening was enlarged, the bone falling into a mass of carious detritus composed of the cancelated structure of the bone. The inside of the jaw, for quite an inch to either side of the hole, was completely broken down. Among the patches removed by the burr, the apex of a tooth root was found.

